# Secondary Benefits to Attentional Processing Through Intervention With an Interactive Maths App

**DOI:** 10.3389/fpsyg.2019.02633

**Published:** 2019-11-26

**Authors:** Nicola J. Pitchford, Laura A. Outhwaite

**Affiliations:** ^1^School of Psychology, University of Nottingham, Nottingham, United Kingdom; ^2^Centre for Education Policy and Equalising Opportunities, Institute of Education, University College London, London, United Kingdom

**Keywords:** mathematics, attention, child development, low-income countries, educational technology

## Abstract

Previous research has shown that a specific interactive app, designed to support the development of early mathematical skills and delivered on hand-held tablets, is effective at raising mathematical attainment in young children in low-and high-income countries. In the countries where this app has been deployed, teachers have consistently reported improved concentration skills in children who have received intervention with this app. To investigate the legitimacy of these claims, we conducted secondary data analyses of children’s performance on core cognitive tasks to examine if additional benefits are observed in children who received intervention with the interactive maths app compared to those that did not. We drew on data from a three-arm randomized control trial conducted in a primary school in Malawi (Pitchford, 2015). In addition to assessing mathematical skills, children’s visual attention, short-term memory, and manual processing speed were examined at baseline, before the introduction of the maths app intervention, and at endline, after the intervention had been implemented for 8 weeks. A group of 318 children (73–161 months) attending Standards 1–3 of a Malawian primary school were randomized to receive either the new maths app (treatment group), a non-maths app that required similar interactions to engage with the software as with the maths app (placebo group), or standard teacher-led mathematical practice (control group). Before and after the 8-week intervention period, children were assessed on mathematics and core cognitive skills. Results showed that the maths app intervention supported significant and independent gains in mathematics and visual attention. Increases in visual attention were attributable only to interactions with the maths app. No significant benefits to attention were found from using the tablet device with non-maths software or standard class-based mathematical practice. These results suggest that high-quality interactive, educational apps can significantly improve attentional processing in addition to the scholastic skills targeted by the intervention.

## Introduction

The United Nations Convention on the Rights of the Child (1989) states that all children have the right to education (Article 28) in order to achieve their full potential (Article 29.1). This includes access to inclusive and equitable quality education, as articulated in the Sustainable Development Goal 4 (United Nations, 2015). However, there are significant challenges to achieving this, particularly in low-income countries, such as Malawi, where school days are short, classrooms are overcrowded and poorly resourced, and teachers are frequently under qualified (Hubber et al., 2016). Consequently, only 40% of all primary school children in Malawi achieve minimum standards in learning mathematics (Chimombo, 2005; Milner et al., 2011). As such, efficient, effective, and evidence-based interventions that can support children’s learning and development and do not rely too heavily on teacher quality could be an effective means of addressing some of the educational challenges faced in Malawi.

An emerging evidence base demonstrates the potential for educational touchscreen applications (apps) to support the development of domain-specific mathematical knowledge (Herodotou, 2018; Xie et al., 2018). In particular, a randomized control trial (RCT) conducted in Malawi found children in the first 3 years of primary school made significantly greater mathematical learning gains when using hand-held touch-screen tablets with an interactive, child-centered maths app, compared to standard teacher-led mathematical practice (Pitchford, 2015). Expansion of this program within Malawi has shown that the same app is also beneficial for children with special educational needs (Pitchford et al., 2018) and girls make just as much progress compared to their male peers (Pitchford et al., 2019). When implemented at the start of primary school, this app can prevent a gender discrepancy in early mathematical attainment from emerging (Pitchford et al., 2019). Similar learning gains in mathematics with the same technology have been found in the UK with children aged 4–7 years (Outhwaite et al., 2017, 2019) and in Brazil with bilingual children aged 5–6 years (Outhwaite et al., under review). This collective evidence base suggests app-based learning can improve domain-specific mathematical skills when implemented in vastly different educational contexts and could be a viable solution to addressing the global learning crisis.

In addition to scientific evidence on effectiveness in mathematics, teachers in the countries where this app has been deployed consistently report secondary benefits in concentration, as children appear to be more focused after using the app. We are in a unique position to evaluate this claim empirically, through secondary data analysis of performance on a range of cognitive skills that were also assessed during the first RCT conducted with this maths app intervention in Malawi (Pitchford, 2015). The cognitive assessment battery included experimental measures of visual attention, short-term memory, working memory, spatial intelligence, manual processing speed, and manual co-ordination that were delivered *via* a specially designed touch-screen assessment app (Pitchford and Outhwaite, 2016). In establishing proof of concept for the assessment app, results showed visual attention, short-term memory and manual processing speed had good test-retest reliability and predictive criterion validity (Pitchford and Outhwaite, 2016). Accordingly, the current study reports secondary data analysis on these domain-general measures across the three arms of the RCT reported by Pitchford (2015).

A substantial body of previous research indicates that visual attention, short-term memory, and processing speed are associated with early mathematical development. In particular, visual attention refers to selective and sustained focus toward visual stimuli (Korkman et al., 1998). It develops rapidly during early childhood (Aunola et al., 2004; Beery and Beery, 2004) and reaches maturity around 10 years (Klenberg et al., 2001). Previous research suggests attention underpins early mathematical development (Duran et al., 2018; Kim et al., 2018) beyond measures of general intelligence (Blair and Razza, 2007) and fine motor skills (Sortor and Kulp, 2003). Brain imaging studies have shown that children aged 8 years demonstrate greater activation in the prefrontal cortex compared to their older peers when completing novel addition and subtraction tasks, suggesting more attentional resources are required when learning new mathematical content compared to when knowledge is automated (Rivera et al., 2005). In the classroom context, strong attentional abilities enable children to focus on and complete the required task (Ruff and Rothbart, 2001; Kolkman et al., 2014). Teachers often use the term concentration to refer to attentional processing.

Short-term memory, the ability to hold information in mind (Kolb and Wishaw, 2009), follows a gradual and linear developmental trajectory that continues into adolescence and adulthood (Luciana et al., 2005; Best and Miller, 2010). In young children, there is a high degree of overlap between short-term and working memory (Aben et al., 2012) with working memory, the ability hold and manipulate information in mind (Miyake et al., 2000), beginning to develop around 4 years of age (Gathercole et al., 2004). A child’s memory capacity is also shown to support early mathematical development (Bull et al., 2008; Peng et al., 2018), accounting for 25% of the variance in mathematical outcomes (Cragg et al., 2017), beyond other cognitive measures of general intelligence (Gathercole and Alloway, 2006; Raghubar et al., 2010). Strong memory skills allow children to hold critical information, such as interim totals in mind, keep track of counting steps, and retrieve number facts from memory (Gathercole et al., 2006; Bull and Lee, 2014).

Processing speed, a central mental capacity (Kail and Salthouse, 1994), also develops rapidly during childhood (Kail, 1991; Anderson et al., 2001) and is associated with mathematical difficulties, such as those observed in children born pre-term (Mulder et al., 2010; Simms et al., 2014). Developmental cascade models suggested in early childhood processing speed is initially closely intertwined with executive functions, such as visual attention and short-term memory (Fry and Hale, 1996; Mulder et al., 2011). However, as children get older executive functions progressively decouple from processing speed and make a strong, unique contribution to mathematical development (Clark et al., 2014).

In addition, longitudinal research has demonstrated a unique and reciprocal relationship between these domain-general cognitive skills and emergent mathematical ability (Welsh et al., 2010; Van der Ven et al., 2012). Furthermore, high-quality, teacher-led mathematics instruction has been shown to have the dual benefit of teaching domain-specific mathematical knowledge and developing domain-general cognitive skills not explicitly targeted by the intervention (Clements and Sarama, 2013; Clements et al., 2016). Expanding evaluations of educational interventions to include domain-general cognitive abilities is vital to understand holistically how the maths app intervention might impact toward achieving a child’s full potential.

The maths app at the focus of this study embodies the Science of Learning principles of active, engaged, meaningful, and socially interactive learning with a specific learning goal (Hirsh-Pasek et al., 2015). Active, minds-on learning in the maths app is fostered through the direct manipulation of virtual objects, verbal labels, and numerical representations (Lindahl and Folkesson, 2012), shown to be supportive of mathematical development in a technological learning environment (Moyer-Packenham and Suh, 2012; Moyer-Packenham et al., 2016). The simultaneous presentation of auditory and visual input is characteristic of multi-sensory learning, which is known to facilitate children’s understanding (Pavio, 1986; Carr, 2012). Engaged learning is supported by immediate feedback (positive and negative) given after every interaction with the maths app and external rewards for correct responses. This app-based, child-centered approach is suggested to support motivational enhancement (Couse and Chen, 2010). Meaningful learning in the app is promoted through staged curriculum content that builds on previous knowledge (Magliaro et al., 2005), increases in level of difficulty, and extends children beyond their current ability level (Vygotsky, 1978; Inal and Cagiltay, 2007). The end-of-topic quizzes assess acquired knowledge and engender retrieval-based practice, shown to improve learning outcomes (Dunlosky et al., 2013; Grimaldi and Karpicke, 2014). Socially interactive learning is also evident in the app through the on-screen teacher providing demonstrations with short, clear task instructions (Troseth et al., 2006).

Overall, many of these app features, specifically, the staged curriculum, contingent feedback, rewards, and the opportunity of deliberate practice are consistent with direct instruction (Kirschner et al., 2006). Meanwhile, the opportunity for self-regulated learning, choice, and control through the child’s in-app profile is characteristic of free play (Gray, 2015). As such, the maths app capitalizes on these benefits of direct instruction and self-regulated play (Naismith et al., 2004; Weisberg et al., 2016) to provide an efficient child-centered and scaffolded learning environment (Mayer, 2004; Mayo, 2009) tailored to individual needs (Slavin and Lake, 2008) enabling individualized and structured instruction (Adams and Carnine, 2003) without additional, time-consuming, and teaching demands (Kucian et al., 2011; Hilton, 2016).

This study addressed two key aims. First, we investigated if the combination of tablet technology with the educational maths app software evaluated in Malawi (Pitchford, 2015) has any additional benefits to cognitive development that extends beyond the scholastic skill of mathematics being targeted by the app-based intervention. Accordingly, this study adds to the data presented in Pitchford (2015) by exploring the relative contributions of the tablet device and the maths app software in supporting the development of core cognitive skills in comparison to standard teaching practice in a Malawian context. Specifically, for each of the three domain-general cognitive skills assessed (visual attention, short-term memory, and processing speed), this study asked do children make more progress with the maths app (Group 1 treatment) compared to the non-maths app (Group 2 placebo) or standard mathematics practice (Group 3 control)? Second, based on the bidirectional hypothesis, which suggests a reciprocal relationship between domain-general cognitive skills and emergent mathematical abilities (Welsh et al., 2010; Van der Ven et al., 2012; Clements et al., 2016), this study examined if any significant gains in domain-general cognitive skill(s) were associated with, or independent of, the learning gains in mathematics (Pitchford, 2015). Examining these research questions in a Malawian context is vital to building a strong evidence-base that can inform education policy and practice on the use of educational maths apps as a means of addressing Sustainable Development Goal 4 (United Nations, 2015).

## Methods

### Design

Secondary data analysis was conducted on a randomized control trial (RCT) that assessed core cognitive skills not analyzed previously, in addition to the main outcome variable, mathematics attainment, that was reported by Pitchford (2015). The secondary data analyses were conducted to examine domain-general cognitive development in response to a math app intervention compared to a non-maths app intervention and standard teacher-led mathematics practice with children aged 6–13 years attending the first 3 years of formal education in Malawi. The RCT was conducted in a medium-sized, urban primary school in Lilongwe, the capital of Malawi during the first 10 weeks of the 2013–2014 academic year. Participating children were randomly allocated to one of three groups; the math app intervention (Group 1 treatment), a non-maths app control (Group 2 placebo) or standard mathematical practice (Group 3 control).

In this design, the placebo group was critical for disentangling the generic effects of using tablet technology from the specific effects of the maths app content used in the treatment group. Furthermore, the placebo group controlled for other extraneous variables that may influence study outcomes. First, the placebo group controlled for potential effects associated with smaller class sizes because both tablet interventions (treatment and placebo groups) were delivered in small groups of 25 children, compared to class sizes of 70–80 children in the standard mathematical practice group (see below). Second, it controlled for potential Hawthorne or novelty effects associated with using the tablet technology because children in the treatment and placebo groups had more exposure to tablet devices, which was also the method used to assess the children on mathematical and cognitive skills before and after the 8-week intervention period.

### Ethics Approval

Ethics approval for the secondary data analysis reported here was not required by the School of Psychology, University of Nottingham, whose ethics board complies with the guidelines of the British Psychological Society. Ethics approval for the original study, on which this secondary data analysis is based, was given by the Ministry of Education, Science and Technology, the school’s Parent Association, and the local Community Chief in Malawi. Due to the high levels of illiteracy in Malawi, it was not possible to gain signed parental consent. Insisting on signed parental consent would have resulted in a biased sample. Accordingly, as is standard practice in Malawi, parents were informed of the study through visual posters displayed in the school and dissemination of study details through the Parent Association and local Community Chief. Opt out consent was applied to parents of participating children. Evaluators gained child assent at the start of each assessment by asking the child if they were happy to play some games. No parent requested that their child did not to participate in the study and no child declined the invitation to take part.

### Malawi Context

World education data highlights Malawi as one of the poorest countries in the world for educational performance; 98% of children do not possess skills beyond basic numeracy (UNESCO-IBE, 2010). Furthermore, the education system in Malawi is ability (Standard) based but also suffers from high repetition and drop-out rates, hence children often repeat years and/or start schooling at a late age. This means the age of children in the Malawi educational system may not correspond to the chronological age in a high-income, Western, educational system. Rather, the Standard the child attends relates to their educational ability so children of different ages can be placed in the same Standard.

Furthermore, in Malawi children’s access to tablet technology is largely limited to education and only in a few schools. Tablet devices were not used in the school where this RCT took place prior to this study commencement and are extremely rare in family homes. As such, the Malawi context represents a unique opportunity to examine empirically the impact of tablet device hardware (e.g., the “iPad”) and software app on child development.

### Participants

**Figure 1:**
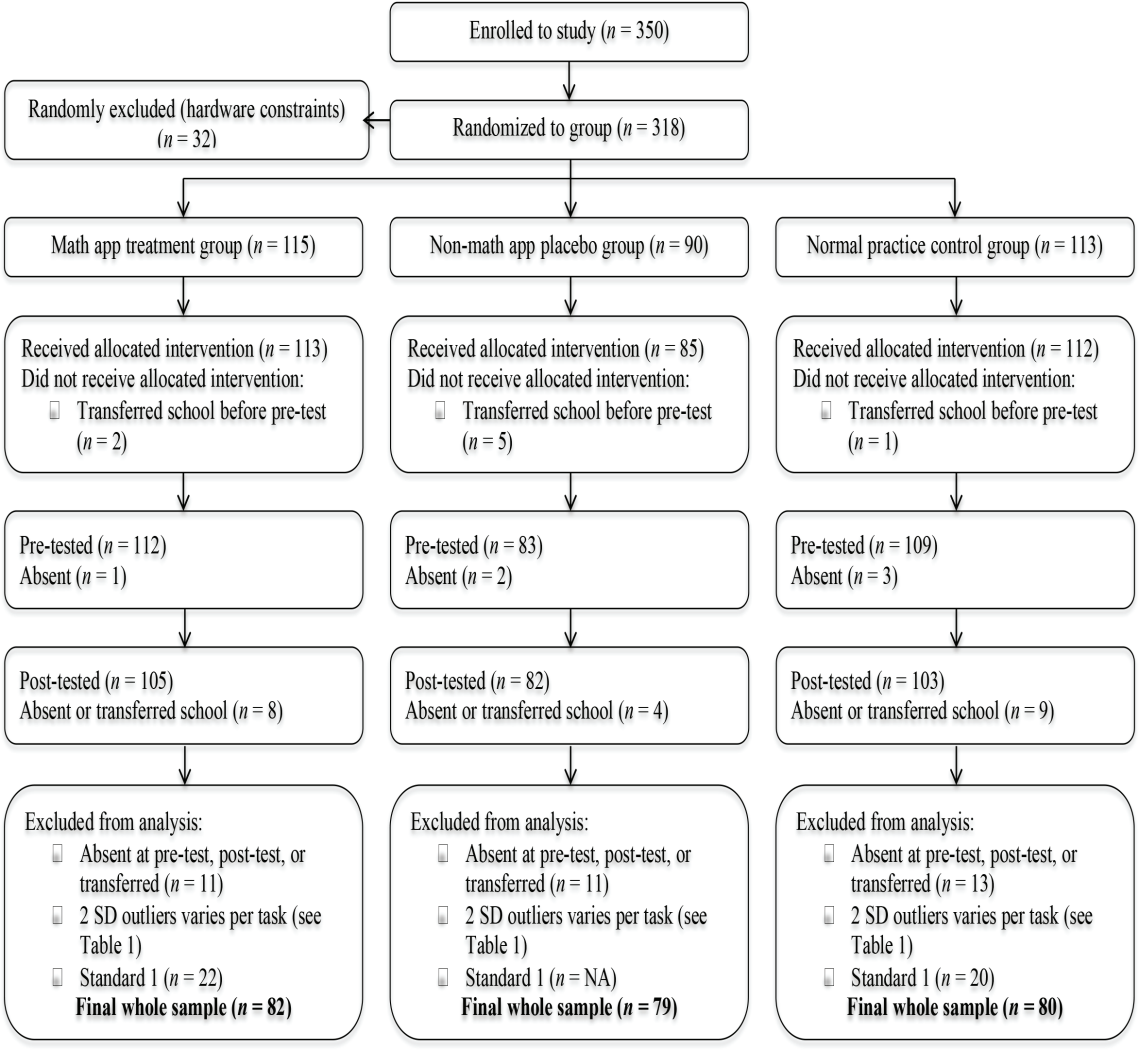
Consort table depicting participant flow through the RCT.

The CONSORT (2010) data in Figure 1 summarizes the study sample at each stage of the RCT. In total, 350 eligible children were enrolled into the study by the first author and two assistants, prior to randomization. Eligibility criteria and sample size were based on school attendance in the first 3 years of primary school (Standards 1–3), during the first 2 days of the 2013–2014 academic year. Any potential learning difficulties or special educational needs for individual children were unknown, as the school did not have details of this for participating children. This is common practice in Malawi where only marked difficulties (e.g., blindness, deafness, mutism, cerebral palsy, and Down syndrome) are typically identified.

Due to hardware constraints restricting the size of the tablet intervention groups (Groups 1 and 2), 32 children were randomly excluded from the study. The remaining 318 children were randomly allocated to one of the three groups. There were 115 children assigned to Group 1 (treatment) and received the maths app intervention. Ninety children were assigned to Group 2 (placebo) and used the non-maths app intervention. Due to smaller class sizes, Standard 1 children were not allocated to Group 2. The remaining 113 children were allocated to Group 3 (control) and received standard teacher-led mathematical practice. Prior to pre-test assessments eight children transferred school and so did not receive their allocated intervention.

A total of 304 children completed the pre-test mathematical and cognitive skill assessments. Six children were absent at pre-test but still received their allocated intervention. Of the 304 children that were pre-tested, 290 completed the post-test assessments; 20 children were absent at post-test or had transferred school during the intervention period. Children were excluded from the final analyses based on the following criteria: (1) children that were absent at pre-test or post-test or had transferred school, (2) children performing two standard deviations or more above and below the group mean for each task (*n* varied per task, see Table 1), and (3) children in Standard 1 (due to smaller class sizes, see above).

**Table 1:** Sample structure including mean (SD) and min-max given for age (months) with gender ratios for final sample and each of the outcome variables per group (±2 SD outliers excluded).

Outcome variables sample structure	Group 1 (maths app treatment)	Group 2 (non-maths app placebo)	Group 3 (standard practice control)
**Whole sample**	***n =* 82**	***n =* 79**	***n =* 80**
Age (months)	98.63 (14.92) 74–161	101.19 (14.80) 79–147	99.26 (14.28) 75–139
Gender (F:M)	40:42	43:36	41–39
**Visual attention**	***n =* 74**	***n =* 66**	***n =* 67**
Age (months)	99.03 (15.51) 74–161	102.23 (15.10) 79–147	101.19 (14.54) 75–139
Gender (F:M)	36:38	35:31	35:32
**Short-term memory**	***n =* 73**	***n =* 68**	***n =* 74**
Age (months)	98.25 (15.03) 74–161	100.37 (13.62) 79–147	99.36 (14.21) 75–139
Gender (F:M)	34:39	37:31	39:35
**Manual processing speed**	***n =* 76**	***n =* 74**	***n =* 73**
Age (months)	98.92 (15.20) 74–161	101.00 (14.71) 79–147	100.32 (14.41) 75–139
Gender (F:M)	37:39	40:34	38:35
**Mathematics**	***n =* 76**	***n =* 71**	***n =* 77**
Age (months)	95.58 (15.44) 74–161	100.14 (14.80) 79–147	98.39 (13.76) 75–139
Gender (F:M)	36:40	40:31	41:36

The final sample consisted of 241 children aged between 6 years, 2 months and 13 years, 5 months in Standards 2–3. This large age range is typical for Malawi’s ability based educational system and reflects inclusion of children who started formal schooling at a late age and/or have been required to repeat grades. Data identifying these children is not available. The final sample structure for each task (with the outliers removed), split by instructional group, including age and gender demographic information is summarized in Table 1.

### Maths App Intervention

Children allocated to receive the maths app intervention (Group 1 treatment) used the maths apps modules: Masamu 1 (Maths in Chichewa), Masamu 2, Count to 10, and Count to 20, on touch-screen tablet devices for a total of 20 h over the 8-week intervention period. The maths app software was developed by *onebillion*, an educational not-for-profit based in the UK,^1^ who were joint winners of the Global Learning XPRIZE. The app focuses on the acquisition of core, basic mathematical skills, including, counting, addition, subtraction, shape, space, and color recognition and aligns with the Malawi National Primary Curriculum for Standards 1–3 (Chirwa and Naidoo, 2014).

**Figure 2:**
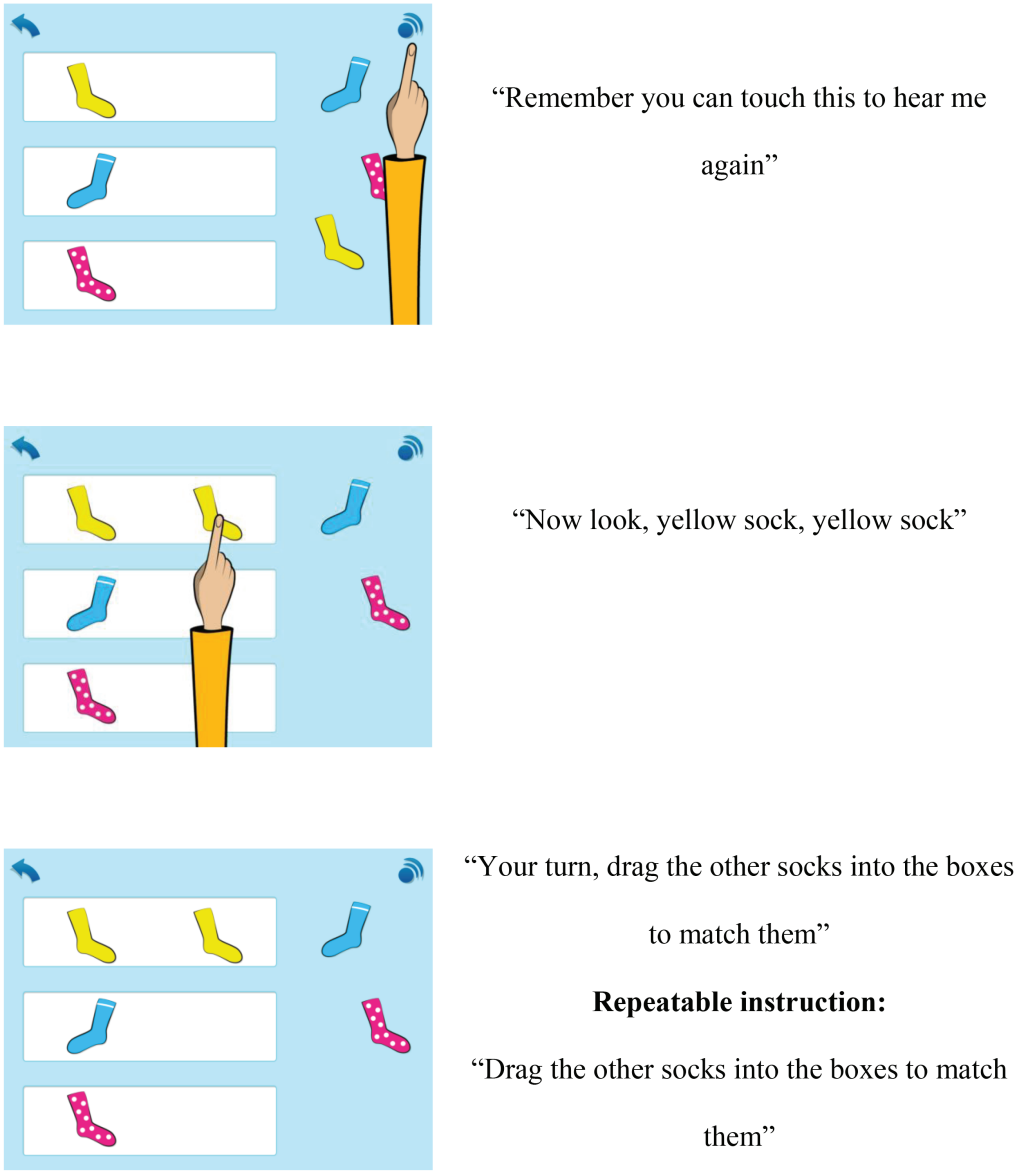
Example item and task instructions for Topic 1, Sorting and Matching with verbal instructions from the maths app intervention (courtesy of *onebillion*).

In this study, the app was delivered in the child’s local language, Chichewa, *via* headphones connected to the tablet device. All participating children across Standards 1–3 progressed through the different topic content covered in the app individually, at their own pace, and in the order presented within the app. Children could choose which modules to work from, but within the different modules activities were highlighted automatically to encourage children to work progressively through the app. They could however switch between modules and activities as they wished. Children also had the opportunity to repeat instructions and/or activities as often as needed. For example, to complete Maths 3–5, Topic 1, Sorting and Matching, children were required to complete seven sets of learning activities focused on sorting and matching different items by type, shape, size, and color, followed by an end of topic quiz. Screenshots of example activity items and verbal instructions for Topic 1 are included in Figure 2 (courtesy of *onebillion*).

The app is grounded in the Science of Learning principles of active, engaged, meaningful, and socially interactive learning with an explicit learning goal (Hirsh-Pasek et al., 2015). For example, the interactive pictures, audio, and animation features with clear objectives and instructions from the virtual teacher (see Figure 2) included in each activity are consistent with the principle of active learning with virtual manipulatives. In line with the principle of engaged learning, formative feedback was given within the app through each interaction the child made. For example, after each correct response during learning activities, children received immediate positive feedback in the form of a visual tick and positive audio. If a child answered incorrectly, they received a negative tone and were encouraged to try again by the in-app teacher who repeated the specific question. Upon successful completion of learning activities in each topic, children also received positive rewards including visual stars and children cheering audio. Other rewards included a certificate upon successful completion of the end of topic quiz.

The end of topic quizzes included 10 questions from the previous learning activities within that topic and were designed to assess children’s knowledge of the mathematical concepts covered, therefore engendering retrieval-based learning and embodying the principle of meaningful learning. Children were required to achieve 100% pass rate on an end of topic quiz included in the app software in order to progress to the next topic. Within each topic, the app was structured to gradually introduce children to the targeted mathematical concept and increase in level of difficulty as children successfully progressed through the content. Between topics, the app curriculum content also builds on children’s prior knowledge. The presence of the in-app teacher who provided clear task demonstrations and instructions, which could be repeated as required by the user, incorporated aspects of socially interactive learning.

### Non-Maths App Intervention

Children assigned to receive the non-maths app intervention (Group 2 placebo) used educational apps focused on musical ability and design: Music Sparkles (*Kids Game Club*©), Drawing Pad (*Darren Murtha Design*©), Toca Tailor, and Toca Hair Salon (both *Toca Boca AB*©), on touch-screen tablet devices for a total of 20 h over the 8-week intervention period. All of the non-maths apps were all freely available in the App Store. These apps were chosen for the placebo group as they are non-verbal and do not teach mathematical concepts covered in the *onebillion* app, but require similar visual discrimination and attention skills (segmentation and selection of objects) and fine manual skills (drag and drop on-screen movements) to interact with the apps. Children were free to choose which apps they worked with in each session and could switch between apps within a session as desired.

### Standard Mathematical Practice

Standard teacher-led mathematical practice followed the Malawi National Primary Curriculum for each Standard and was delivered daily by class teachers in Chichewa to larger groups of 70–80 children inside the regular classroom. Typical lessons consisted of mathematical questions written on the chalkboard or dictated orally that children were required to complete in their notebooks. Standard mathematical lessons typically lasted 1 h and were delivered on average 2–3 times a week. The content of the maths app intervention developed by *onebillion* was closely aligned to the content covered in the Malawi National Primary Curriculum, so children in the control group should have received similar instructional content delivered by class teachers as the children who received the maths app intervention. However, children in the treatment group could work through the app content at their own pace, whereas for children in the control group, pace of delivery was determined by the class teacher. Accordingly, it is likely that children receiving the maths app intervention could access a broader range of maths content than children receiving standard classroom practice.

### Mathematical and Cognitive Skill Assessments

**Figure 3:**
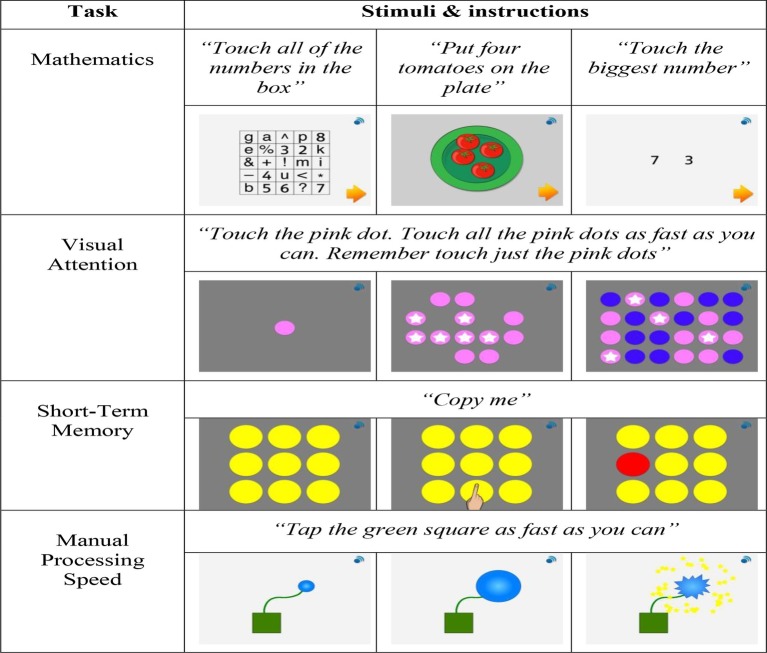
Schematic illustration of the tasks used to assess mathematics and core cognitive skills (adapted from Pitchford and Outhwaite, 2016).

Children’s mathematical and cognitive skills were assessed using an assessment app designed especially for this study by the first author and programmed by *onebillion*. The assessment app included a battery of tasks designed to assess the scholastic skill targeted by the intervention (mathematics) and domain-general ability (visual attention, short-term memory, working memory, spatial intelligence, manual processing speed, and motor co-ordination). These measures of core cognitive and fine motor skills were chosen based on their close association with the development of mathematics (Berg, 2008; Mulder et al., 2010; Westendorp et al., 2011; Simms et al., 2014; Pitchford et al., 2016). The tasks were operationalized to address issues of construct bias (Grigorenko et al., 2001) and comparative research in Malawi and the UK found the assessment app to be cross-culturally valid (Pitchford and Outhwaite, 2016). However, in the Malawi sample, adequate levels of test re-test reliability for the assessment tasks were only seen for the measures of mathematics, visual attention, short-term memory, and manual processing speed (Pitchford and Outhwaite, 2016). As such, the current study only analyzed data for these measures. Illustrations of each task included in the current study are given in Figure 3.

#### Visual Attention

Visual attention was assessed with a speeded search task (Pitchford et al., 2011). Before each of three experimental trials, children were presented with a baseline practice trial in which they were shown a single colored dot, followed by an array of either 8, 12, or 16 same colored dots. Children were instructed to touch the dots as fast as possible. In the experimental trials, children were required to distinguish and touch all the colored dots presented in the practical trial from a display of different colored distractor dots. For each trial, time taken to complete the baseline trial was subtracted from the time taken to complete the experimental trial, thus generating a measure of visual attention that was not confounded by manual processing speed. An overall measure of visual attention was derived from the mean response times taken to complete the three experimental trials. This visual attention task demonstrated good test-retest reliability (*r =* 0.42) and predictive criterion validity (*r* = −0.34; Pitchford and Outhwaite, 2016).

#### Short-Term Memory

A forward spatial span task was used to assess short-term memory (Brunetti et al., 2014). Children were presented with a three-by-three grid of yellow circles. The virtual instructor demonstrated the pattern to be recreated by the child by touching the yellow circles. When the demonstrator touched a yellow circle it turned red, momentarily, until the demonstrator touched the next circle in the sequence. Children were then required to repeat the order they had been presented. The number of circles in the sequence increased in line with progression through the test; starting at one and increasing to nine. The task discontinued after three successive incorrect trials. An overall measure of short-term memory was indicated by the raw number of trials completed correctly. This short-term memory task demonstrated moderate test-retest reliability (*r* = 0.34) and predictive criterion validity (*r* = 0.21; Pitchford and Outhwaite, 2016).

#### Processing Speed

A single-finger-tapping task was used to assess manual processing speed (Witt et al., 2008). Using the index finger on their dominant hand children were required to tap a green box displayed on the touch-screen continually, as fast as they could, which caused a blue balloon to increase in size. The task was complete when the child had tapped the green box 30 times causing the balloon to pop. An overall measure of manual processing speed was calculated from the mean completion time across the two trials. This processing speed task demonstrated moderate test-retest reliability (*r* = 0.35) and predictive criterion validity (*r* = −0.23; Pitchford and Outhwaite, 2016).

#### Mathematics

A test consisting of 98 items, measuring different aspects of curriculum and conceptual knowledge was used to assess mathematics. The curriculum questions were based on the content of the *onebillion* maths apps (Pitchford, 2015) that are grounded in the UK national curriculum, and covered topics such as counting, addition, subtraction, and shape and space recognition. The mathematics curriculum in Malawi is based on the UK curriculum and places a strong focus on the acquisition of numeracy skills (Chirwa and Naidoo, 2014). The conceptual questions were based on the Early Grade Mathematics Assessment (EGMA; Brombacher, 2010) and the Numerical Operations subtest of the WIAT-II (Wechsler, 2005; see Pitchford, 2015). Concepts assessed included symbolic understanding, numbers in relation to each other, number line understanding, counting, number sense (quantity estimation), simple and complex addition and subtraction, multiplication, and division. Task difficulty increased in line with task progression and discontinued after three successive incorrect answers. An overall mathematics score was determined from the total number of questions answered correctly. This mathematics task demonstrated strong test-retest reliability (*r = 0*.73; Pitchford and Outhwaite, 2016).

### Procedure

#### Group Allocation

Randomization to Group was conducted prior to pre-test assessments using a computer program written by *onebillion* and occurred across Gender and Class. At the Gender level, this procedure maximized an equal gender representation in each group. At the Class level, this procedure controlled against potential teacher effects across different classes, which was particularly important for the internal validity of the study as teaching staff implemented the app interventions (Groups 1 and 2), in order to maximize external validity. The same teachers that implemented the app interventions also delivered standard mathematical instruction in the usual classroom setting (Group 3). The research team remained independent from the intervention implementation and standard mathematical practice. The evaluators were also blind to condition of individual children at both pre-test and post-test.

#### Intervention Implementation

The app-based interventions (Groups 1 and 2) were implemented in small groups of 25 children in a purpose-built Learning Centre during the school day. The Learning Centre was a small classroom that housed all of the intervention equipment and had an independent solar-powered electricity supply used to charge the tablets over night and was located within the school grounds.

The intervention period lasted for 8 weeks (40 school days). On alternative days, class teachers implemented the maths app intervention (Group 1) and the non-maths app intervention (Group 2). Children allocated to Groups 1 and 2 used their assigned intervention for 1 h on each day of use, totaling 20 h of intervention exposure. The class teachers overseeing the implementation of the study established the timetable for intervention exposure and assigned one group of teaching staff to the maths app intervention (Group 1 treatment) and a different group of teaching staff to the non-maths app intervention (Group 2 placebo). As such, teachers were aware of the study design and of children’s group allocation. However, teachers were not involved in assessing the children’s mathematical and cognitive skills before and after the 8-week intervention period; this was conducted by the independent research team who were blind to group allocation of participating children.

In each Standard, the maths app intervention (Group 1 treatment) was implemented while the other two groups received standard mathematical instruction to equate total time spent learning mathematics as closely as possible. The non-maths app intervention was implemented during a non-maths session, as determined by the teachers administering the trial, so that it would fit into the daily timetable with minimal disruption to the teaching of key skills. The timetable for the intervention exposure was organized by the class teachers overseeing the implementation of the study.

In both app intervention groups (Groups 1 and 2), children accessed their assigned software on iPad minis. These devices were chosen based on their suitable size for young children to use and the good battery life. The children used the tablet devices while seated on the floor on bamboo mats in the Learning Centre. *Onebillion* provided 50 iPad minis for the duration of the study. This enabled 25 iPads to be used on alternate days, as per the intervention exposure timetable, while the other 25 iPads were charged. The class teachers were responsible for ensuring the tablet devices were fully charged for the school day. To ensure children accessed the correct software for their allocated group, the iPad minis were color coded (Group 1 treatment, red; Group 2 placebo, blue). In the maths app intervention group, children were also given their own profile within the *onebillion* maths app. To ensure children accessed the correct profile, the iPad minis were labeled with the child’s photograph and study ID number. At the end of the study, the participating school continued to have access to the tablet device hardware and maths app software, so the apps were available to all children.

#### Implementation Support and Monitoring

A volunteer from the Voluntary Services Overseas (VSO) in Malawi provided additional technical support for using the technology. Teachers were trained by the maths app developers, *onebillion*, on how to use the tablets and apps (both maths and non-maths apps) prior to study commencement. The role of the teachers and volunteer in delivering the intervention focused on technical support, such as ensuring children were using the tablet allocated to them, and behavior management, such as ensuring children were on task. No measures of adherence and compliance were obtained systematically throughout the trial due to practical constraints.

#### Assessment Administration

The tablet-based assessments of mathematical and cognitive skills were administered immediately before (1 week) and immediately after (1 week) the 8-week intervention period using the specifically designed assessment app (Pitchford and Outhwaite, 2016). The assessments were delivered on the same hand-held tablets as were used in the intervention. Tablet technology was chosen as it enabled large groups of children to be objectively assessed within a short time period.

The tablet-based assessments were conducted in groups of up to 50 children by the first author and two assistants in the Learning Centre. The evaluators were blind to group allocation of individual children. The tasks were presented in the order outlined in Figure 3. Individual tasks were demonstrated visually to the children by the first author at the start of each task. Task instructions were delivered in Chichewa, the child’s local language, *via* headphones connected to the tablets. The children completed the tasks independently and could repeat task instructions if needed by pressing a small button in the corner of the screen. Class teachers and the volunteer from VSO supervised the group administration of the assessments in one 45-min session per group, providing additional language support for the children and the first author when needed. Performance data for individual children were recorded by the assessment app and later retrieved through an internet server hosted by *onebillion.*

## Results

Table 2 reports the group mean performance on each of the cognitive and mathematics measures at pre-test and post-test, as well as mean gain scores (post-test minus pre-test) for each of the three groups. For mathematics, data from Pitchford (2015) were collapsed across Standard and Gender and re-analyzed at the Group level. To account for pre-test differences in the outcome variables across the three groups (see Table 2), difference score-based analyses were considered the most appropriate for the current study (Van Breukelen, 2006; Thomas and Zumbo, 2012).

**Table 2:** Descriptive data for each outcome variable across the three groups.

Outcome variables	Group 1 (maths app treatment)	Group 2 (non-maths app placebo)	Group 3 (standard practice control)
**Visual attention (s)**	***n =* 74**	***n =* 66**	***n =* 67**
Pre-test mean (SD)	0.92 (0.29)	0.83 (0.19)	0.89 (0.23)
Post-test mean (SD)	0.64 (0.15)	0.74 (0.13)	0.78 (0.14)
Gain score mean (SD)	−0.28 (0.26)	−0.09 (0.18)	−0.11 (0.21)
Within-group effect size (*d*, 95% CI)	1.21 (0.72–1.71)	0.55 (0.06–1.05)	0.58 (0.09–1.07)
**Short-term memory (/28)**	***n =* 73**	***n =* 68**	***n =* 74**
Pre-test mean (SD)	3.53 (1.80)	3.56 (1.76)	3.62 (1.63)
Post-test mean (SD)	3.99 (1.71)	4.25 (1.66)	4.11 (1.88)
Gain score mean (SD)	0.45 (2.06)	0.69 (2.21)	0.49 (1.80)
Within-group effect size (*d*, 95% CI)	0.26 (−0.20–0.73)	0.40 (−0.08–0.88)	0.28 (−0.18–0.74)
**Manual processing speed (s)**	***n =* 76**	***n =* 74**	***n =* 73**
Pre-test mean (SD)	8.65 (1.83)	8.29 (1.90)	8.35 (2.00)
Post-test mean (SD)	7.72 (1.42)	7.74 (1.26)	7.78 (1.58)
Gain score mean (SD)	−0.93 (1.92)	−0.55 (1.91)	−0.57 (2.04)
Within-group effect size (*d*, 95% CI)	0.57 (0.11–1.03)	0.34 (−0.12–0.80)	0.32 (−0.15–0.78)
**Mathematics (/98)**	***n =* 76**	***n =* 71**	***n =* 77**
Pre-test mean (SD)	20.41 (12.54)	22.89 (12.84)	19.67 (11.11)
Post-test mean (SD)	44.84 (16.37)	36.60 (15.32)	32.22 (15.51)
Gain score mean (SD)	24.43 (12.49)	13.71 (11.29)	12.55 (10.54)
Within-group effect size (*d*, 95% CI)	1.68 (1.15–2.20)	0.97 (0.48–1.46)	0.93 (0.46–1.40)

### Mathematics Gains

To examine the relative contributions of instruction, tablet device, and app software on mathematical gains, a one-way Analysis of Variance (ANOVA) was conducted for gain scores on mathematics ability across Group. Results showed significant Group differences for gains in mathematics, *F*(2, 221) = 24.67, *p* < 0.001. *Post-hoc*, independent samples *t*-test and between-groups effect sizes (Cohen’s *d* with 95% CI; Trafimow, 2015) showed children in Group 1 (maths app treatment group) made significantly greater gains in mathematics compared to Group 2 (non-maths app placebo group) *t*(145) = 5.45, *p* < 0.001, *d = 0*.90 (95% CI = 0.56–1.24), and Group 3 (standard practice control group), *t*(151) = 6.36, *p* < 0.001, *d =* 1.03 (95% CI = 0.69–1.37). No significant difference in mathematical gains was observed between Group 2 (placebo) and Group 3 (control), *t*(146) = 0.65, *p* = 0.520. As expected, this analysis of mathematical gains at the Group level, when data were collapsed across Standard and Gender, replicates the findings reported in Pitchford (2015).

### Cognitive Gains

To examine the relative contributions of instruction, tablet device, and app software on cognitive gains, separate one-way ANOVAs were conducted for gain scores on each cognitive ability assessed. Results showed significant group differences for gains in visual attention only, *F*(2, 204) = 15.91, *p* < 0.001. *Post-hoc*, independent samples *t*-tests and between-groups effect sizes (Cohen’s *d* with 95% CI) showed children in Group 1 (maths app treatment group) made significantly greater gains in visual attention compared to Group 2 (non-maths app placebo group), *t*(138) = 4.95, *p* < 0.001, *d* = 0.84 (95% CI = 0.50–1.19), and Group 3 (standard practice control group), *t*(139) = 4.32, *p* < 0.001, *d* = 0.72 (95% CI = 0.38–1.06). No significant difference in attentional gains was observed between Group 2 (placebo) and Group 3 (control), *t*(131) = 0.40, *p* = 0.691.

No significant Group differences in gains were observed for short-term memory, *F*(2, 220) = 0.28, *p* = 0.753 or manual processing speed, *F*(2, 220) = 0.89, *p* = 0.412.

### Independence of Effects

A Spearman’s Rho correlation analysis showed visual attention was significantly associated with mathematics across the whole sample at pre-test, *r^s^* = −0.29, *p* < 0.001. Given this relationship (Sortor and Kulp, 2003; Blair and Razza, 2007; Duran et al., 2018; Kim et al., 2018) and the ANOVA results reported above, which identified significant visual attention gains in response to the maths app intervention (Group 1 treatment), further Spearman’s Rho correlation analyses were conducted to examine if the observed gains in visual attention for Group 1 (treatment) were independent from their gains in mathematics (Pitchford, 2015). Results showed no significant relationship between gains in visual attention and mathematics in response to the maths app intervention (Group 1 treatment), *r^s^* = 0.08, *p* = 0.527.

## Discussion

Previous research has demonstrated the effectiveness of a specific educational maths app for improving domain-specific mathematical skills in Malawi (Pitchford, 2015; Pitchford et al., 2018, 2019), the UK (Outhwaite et al., 2017, 2019) and Brazil (Outhwaite et al., under review). Alongside this empirical evidence, teachers have anecdotally reported secondary benefits to children’s attention after using the maths app intervention. In response to these claims, this study reports the first empirical evidence that disentangles and evaluates the impact of using hand-held tablet technology from app software content on child development outcomes. Specifically, secondary data analysis from a three-arm RCT examined the relative contributions of the tablet device and the educational maths app software (Group 1 treatment) in comparison to non-maths app software (Group 2 placebo) and standard teaching practice in Malawi (Group 3 control) for supporting the development of domain-general cognitive abilities beyond the domain-specific mathematical skills targeted by the intervention. The current findings are of particular significance for further understanding the impact of educational technologies on child development. It further emphasizes the importance of focusing on the app content over the tablet device alone (Falloon, 2013; Blum-Ross and Livingstone, 2016).

### Improvements in Visual Attention

In addressing the first aim, this study found children who received the maths app intervention (Group 1 treatment) made significantly greater gains in visual attention compared to their peers who used the non-maths app intervention (Group 2 placebo; between-groups effect size = 0.84) or standard teacher-led mathematics practice (Group 3 control; between-groups effect size = 0.72). Gains in visual attention for children in Group 1 (treatment) were characterized by a large within-group effect size of 1.21 compared to the medium within-group effect sizes observed for Group 2 (placebo; *d* = 0.55) and Group 3 (control; *d* = 0.57). These significant improvements in visual attention in response to the educational maths app adds to the gains in domain-specific mathematical knowledge previously reported (Pitchford, 2015) and replicated here, when data were collapsed across Standard and Gender, and analyzed at the Group level. Furthermore, this empirical evidence corroborates anecdotal teacher observations of intervention implementation which reported greater focused attention (concentration) in the classroom after using the maths app.

In addressing the second aim, results showed baseline visual attention and mathematical performance were significantly correlated; children with faster visual search skills also had stronger mathematical skills. Given this observed relationship, which is also consistent with previous research demonstrating an association between the two skills (Sortor and Kulp, 2003; Blair and Razza, 2007; Duran et al., 2018; Kim et al., 2018), further analyses were conducted to examine if the gains in visual attention observed for Group 1 (maths app treatment group) were reflective or independent of gains in mathematics. Results showed no significant correlation between gains in visual attention and gains in mathematics in response to the maths app intervention (Group 1 treatment). This suggests that children’s improvements in visual attention in response to the maths app intervention were independent of mathematical learning gains. This evidence corroborates previous research demonstrating high-quality mathematics instruction can have “spill-over” benefits to domain-general cognitive skills, on top of the domain-specific mathematical knowledge targeted by the intervention (Clements and Sarama, 2013; Clements et al., 2016).

This study also has implications for current debate about screen time and child development. Previous research has shown no association between screen time with television and later attentional deficits, as reported by parents (Zimmerman and Christakis, 2007). However, in the current study, children actively engaged and interacted with the maths app rather than passively being exposed to screen time. Specifically, the interactive, multi-sensory learning environment provided by the maths app may have provided attentional anchors that may have guided children’s action and perception, which together with the multi-touch nature of the tablet device may have allowed children to dynamically engage with the new mathematical concepts (Duijzer et al., 2017). As such, this high level of attentional processing of the mathematical content required to progress within the maths app may have contributed to the observed, secondary benefit of an increase in core attentional skills.

Furthermore, although the apps used in the placebo group also focused on visual discrimination, attention skills and included many features consistent with active (e.g., direct manipulation of virtual objects in a multi-sensory environment) and engaged (e.g., feedback and rewards) learning (Hirsh-Pasek et al., 2015), these apps did not follow a meaningful curriculum and did not include an explicit learning goal as the maths app used in the treatment group did (see above). As children in the placebo group did not demonstrate the same rate of development for attentional skills as the treatment group, this study suggests that the inclusion of well-defined pedagogy and learning goals are underpinning features driving the success of educational apps in supporting the development of domain-specific mathematical and domain-general attentional skills.

These additional, independent benefits are of importance as attentional skills are critical for scholastic development. Classroom activities require children to maintain, sustain, and shift their attention (McClelland et al., 2007; Kent et al., 2014), so instructional practices that improve attentional abilities can play a vital role in supporting the academic success of all children (Rhoades et al., 2011). The current results corroborate teacher reports that the maths app encourages children to follow instructions in class. Furthermore, attentional skills are important in higher-level mathematical processing (Hohol et al., 2017). This reciprocal relationship (Clements et al., 2016) suggests there may be potential long-term, additional benefits to using the maths app in early education, as improvements in attentional skills will also impact on later mathematical ability.

### Short-Term Memory and Processing Speed

In contrast, there were only minimal increases in short-term memory and manual processing speed (see Table 2) and there were no significant differences across the three instructional groups. These results corroborate previous research that found no significant improvements in memory ability following domain-specific mathematical instruction (Messer et al., 2018) or domain-general memory training (Melby-Lervåg and Hulme, 2013; Roberts et al., 2016). Collectively, this evidence suggests memory is challenging to intervene, as improvements to memory capacity typically require changes to basic information processing (Melby-Lervåg et al., 2016), which were also not observed in the current study. Furthermore, improvements to memory and processing speed may require a longer intervention period (Messer et al., 2018). For example, further studies suggest that memory capacity follows a longer, more gradually developmental trajectory (Gathercole et al., 2004), which is beyond the 8-week intervention period implemented in the current study.

### Limitations and Future Directions

While the current study makes a valuable contribution to an emerging evidence based on the impact of educational touchscreen apps on child development (Herodotou, 2018; Xie et al., 2018), five issues should be considered in directing future research. First, it is important to recognize that this study has been conducted in Malawi, where children’s access to tablet technology is largely limited to education and only in a few schools. Tablet devices were not used in the school where this study took place prior to this study commencement and are extremely rare in family homes in Malawi. Therefore, future research examining the impact of screen time with tablet technology content on key areas of child development might benefit from being to be conducted in a context where technology access is more ubiquitous so as to add to the initial evidence reported here (e.g., Bedford et al., 2016). Replicating this study in a high-income country context with digital native children would also afford interesting cross-cultural comparisons and would elucidate the generalizability of our findings.

Second, in the current study, no data were systematically collected for adherence and compliance rates across the different instructional groups. Without this data, it is possible that the effects of the instruction delivered to each of the three groups might be diluted somewhat by inclusion of children who did not reach a particular adherence and compliance threshold. Nevertheless, the observed results point to the importance of the app content over the tablet device (Garrison et al., 2011; Blum-Ross and Livingstone, 2016) in enhancing mathematics and attentional skills, although it is possible that adherence and compliance might have been greater in the maths app treatment group than the non-maths app placebo and standard practice control groups. Future studies should obtain adherence and compliance data so as to investigate this issue.

Third, the current study focused on immediate gains in mathematical and cognitive skills following the maths app intervention; sustained gains assessed through a delayed post-test were not considered. Further longitudinal studies are needed in both low and high-income country contexts, to establish the long-term impact of using educational touchscreen apps on later scholastic attainment and the relative associations with cognitive development. This will help to address potential novelty effects related with app-based mathematics instruction implemented for a short duration (Lieberman et al., 2009). Longitudinal studies are also necessary before a meaningful cost-benefit analysis can be conducted, which is particularly relevant in a low-income country context like Malawi.

Fourth, although random allocation to Group occurred across Gender and Class to maximize equal gender representation and control against potential teacher effects (see above), it is important to acknowledge that class sizes in which the interventions were implemented were uneven; 25 children per class in Group 1 (treatment) and Group 2 (placebo) and 70–80 children per class in Group 3 (control). While this reflects the practical constraints of the study setting and available hardware, these differences may present a confounding factor, potentially impacting the internal validity of the current study findings. However, class size was equal for both of the groups receiving tablet-based interventions, yet only the treatment group (Group 1) showed significant gains in mathematics and visual attention over time. Performance of the placebo group (Group 2), where class size was 25, did not differ significantly to controls (Group 3), where class size was 70–80 children. Thus, it is unlikely that differences in class size are a contributing factor influencing results; however, in building on this initial evidence, future research, where possible, should attempt to ensure equal (or as close to equal) group sizes.

Finally, it is important to acknowledge that the pre and post-test assessments were conducted using the same touch-screen tablet technology hardware as the interventions implemented in Group 1 (treatment) and Group 2 (placebo). This assessment method was chosen based on the practical ease of delivery in the current study context and the lack of relevant and accessible assessment tools appropriate for use in low and middle-income countries (LMICs; Pitchford and Outhwaite, 2016). However, this assessment delivery may present a potential threat to internal validity based on practice and familiarity effects with the hardware devices that may have favored Groups 1 and 2 over Group 3 (control), who had no exposure to the technology. To address this potential confound, in the original RCT of the maths app intervention reported here we administered a paper-based assessment of mathematics curriculum knowledge at post-test (Pitchford, 2015). The same pattern of results was found for both paper-based and digital assessments of mathematics curriculum knowledge, in that only Group 1 (treatment), who received the maths app intervention, showed significant gains in mathematics and visual attention, despite Group 2 (placebo) engaging with the touch-screen tablet hardware for the same amount of time. This demonstrates that practice and familiarity effects with the hardware devices is not likely to be a limiting factor; however, future research could include independent measures of child development and learning that have recently been developed and validated for use in LMICs (Boggs et al., 2019). The inclusion of these additional measures will also help to address the moderate psychometric properties (see above) of the current cognitive tasks.

## Conclusion

Overall, this study shows there were additional benefits to visual attention in response to the maths app intervention (implemented for around 20 h) compared to the non-maths app placebo group and the standard mathematical practice control group. These improvements in visual attention were independent from mathematical learning gains. This evidence suggests, for low-income countries like Malawi, with a history of poor child development and impoverished primary education (Hubber et al., 2016), the use of high-quality educational app content in primary schools can be beneficial in supporting domain-specific and domain-general aspects of child development. Longitudinal studies are required to evaluate the long-term impact of this education technology in relation to a cost-benefit analysis of implementing these high-tech solutions in low-income countries.

## Data Availability Statement

The datasets generated for this study are available on request to the corresponding author.

## Ethics Statement

The studies involving human participants were reviewed and approved by Malawi Ministry of Education, Science and Technology. Written informed consent from the participants' legal guardian/next of kin was not required to participate in this study in accordance with the national legislation and the institutional requirements.

## Author Contributions

NP designed the study and materials, conducted the study in Malawi, processed the data, supervised the analyses, and edited the article. LO conducted the secondary data analyses and wrote the article.

### Conflict of Interest

The authors declare that the research was conducted in the absence of any commercial or financial relationships that could be construed as a potential conflict of interest.

## Footnotes

^1^www.onebillion.org
